# Diagnostic value of calcitonin for lateral cervical lymph node metastasis in medullary thyroid carcinoma: a systematic review and meta-analysis

**DOI:** 10.3389/fonc.2026.1789486

**Published:** 2026-04-02

**Authors:** Li Zhang, Shuangyan Wang, Xun Ye, Xinbin Lin

**Affiliations:** Department of Surgery, The Affiliated Wenling Hospital of Wenzhou Medical University, Wenling, Zhejiang, China

**Keywords:** calcitonin (CTN), diagnostic performance, lateral cervical lymph node metastasis (LLNM), medullary thyroid carcinoma (MTC), systematic review and meta-analysis

## Abstract

**Objective:**

To evaluate the diagnostic performance of preoperative serum calcitonin (Ctn) in detecting lateral cervical lymph node metastasis (LLNM) in patients with medullary thyroid carcinoma (MTC), identify the optimal threshold, and provide evidence for individualized neck dissection strategies.

**Methods:**

Relevant Chinese and English databases, including PubMed, Embase, Web of Science, the Cochrane Library, CNKI, WanFang, and VIP, were systematically searched. Eight studies meeting the inclusion criteria, comprising a total of 951 patients, were selected. The QUADAS-2 tool was employed to assess the risk of bias. Meta-analysis was conducted using R software, comparing preoperative Ctn levels between LLNM and non-LLNM groups, with subgroup analyses performed according to different cut-off values.

**Results:**

Preoperative Ctn levels were significantly higher in the LLNM group compared to the non-LLNM group (SMD = 1.00, 95% CI: 0.50–1.49). At a cut-off value of ≥300 pg/mL, the pooled sensitivity was 0.90, specificity 0.62, and diagnostic odds ratio (DOR) 13.48. At a cut-off of 200 pg/mL, sensitivity was 0.83 and specificity 0.42. The differences in specificity and DOR between the two cut-off groups were statistically significant. The pooled area under the summary receiver operating characteristic (SROC) curve was 0.894.

**Conclusion:**

Preoperative serum Ctn demonstrates excellent predictive performance for LLNM in MTC. A threshold of ≥300 pg/mL serves as an ideal cut-off, providing a reliable, non-invasive indicator to guide “risk-oriented” individualized neck dissection and supplementing the limitations of conventional imaging in detecting micro-metastases.

## 1 Introduction

Medullary thyroid carcinoma (MTC) is a distinct neuroendocrine malignancy originating from the thyroid’s parafollicular cells, or C cells, which are dedicated to the synthesis and secretion of calcitonin (1). Unlike the more common differentiated thyroid cancers, MTC demonstrates a more aggressive clinical behavior, with patient prognosis correlating strongly with disease stage at diagnosis—particularly the extent of lymph node involvement (2). Lateral lymph node metastasis (LLNM), most frequently involving levels II through V, is a common pathway of dissemination in MTC and a major contributor to persistent or recurrent disease following initial surgery (3). The presence of LLNM plays a decisive role in determining the extent of primary surgical intervention, most notably whether a lateral neck dissection is warranted. Moreover, nodal status profoundly influences the postoperative normalization kinetics of key tumor biomarkers, namely calcitonin and carcinoembryonic antigen (CEA). Consequently, the presence of LLNM serves as a major prognostic determinant, directly impacting long-term outcomes and the likelihood of achieving sustained disease-free survival for the patient (4). Consequently, the accurate pre-operative assessment of lateral neck nodal status is paramount for formulating individualized and precise initial treatment strategies.

Current pre-operative evaluation of cervical lymph nodes relies predominantly on imaging modalities, including neck ultrasonography (US), computed tomography (CT), magnetic resonance imaging (MRI), and fluorodeoxyglucose positron emission tomography/computed tomography (18F-FDG PET/CT) (5). However, the sensitivity and specificity of these imaging techniques are limited, particularly in detecting nodal micro-metastases or metastases in lymph nodes without discernible morphological alterations in MTC. Calcitonin (Ctn), a polypeptide hormone specifically secreted by thyroidal C cells, serves as the most sensitive and specific serum tumor marker for MTC (6). The level of calcitonin secretion from the primary tumor is postulated to correlate with both tumor burden and invasive potential (7, 8). Therefore, the potential utility of pre-operative basal serum calcitonin level as a non-invasive biomarker for predicting lateral cervical lymph node metastasis has been a persistent focus of clinical investigation.

This study aims to synthesize and analyze the diagnostic accuracy of Ctn for LLNM, while exploring heterogeneity across different proposed thresholds, thereby aiming to provide high-level evidence to inform evidence-based and individualized neck dissection strategies.

## 2 Methods

### 2.1 Study design

This investigation was conducted as a systematic review and diagnostic test accuracy (DTA) meta-analysis. Its objective was to quantitatively evaluate the diagnostic performance of pre-operative Ctn for predicting LLNM in MTC. The reporting of this study adhered rigorously to the PRISMA-DTA guidelines to ensure methodological transparency and completeness.

### 2.2 Literature search strategy

A systematic and comprehensive literature search was conducted across major electronic databases in both English and Chinese. The sources searched included PubMed, Embase (via Ovid), Web of Science, the Cochrane Library, the China National Knowledge Infrastructure (CNKI), WanFang Data, and the VIP Chinese Science and Technology Periodical Database. The search period covered from database inception to December 1, 2025. To ensure comprehensive retrieval, the search strategy employed a combination of controlled vocabulary (e.g., Medical Subject Headings [MeSH]) and free-text terms, structured around three core components: P (Population): Medullary Thyroid Carcinoma; I (Index Test): Calcitonin; and T (Target Condition): Lateral Cervical Lymph Node Metastasis. The search strategy used for PubMed, for example, was as follows:

(“medullary thyroid carcinoma”[tiab] OR “medullary thyroid cancer”[tiab] OR “MTC”[tiab]) AND (“Calcitonin”[Mesh] OR “calcitonin”[tiab] OR “Ct”[tiab]) AND (“Lymphatic Metastasis”[Mesh] OR “lymph node metastas”[tiab] OR “lateral neck”[tiab] OR “cervical lymph node”[tiab]).

Corresponding Chinese translated keywords, such as “甲状腺髓样癌”, “降钙素”, and “侧颈淋巴结转移”, were utilized for searches within Chinese databases. A supplementary manual examination of the bibliographies from all primary studies incorporated in this analysis, as well as from pertinent review articles, was undertaken to locate any potentially eligible publications that might have been missed during the electronic database searches. No language restrictions were imposed during the search process.

### 2.3 Inclusion and exclusion criteria

Study types: Prospective or retrospective diagnostic accuracy studies, cohort studies, or cross-sectional studies from which data to construct a 2x2 contingency table could be extracted or derived were considered.
Participants: The review included studies involving patients with a histopathologically confirmed diagnosis of MTC who subsequently underwent cervical lymph node dissection. A key requirement was that studies explicitly reported pathological assessment outcomes specifically for lateral cervical lymph nodes.
Index test: The index test of interest was the pre-operative serum calcitonin level (basal or stimulated). Variations in assay methodologies across studies were permitted.
Reference standard: Histopathological examination following therapeutic or prophylactic lateral neck lymph node dissection served as the reference standard for diagnosing lymph node metastasis.
Outcome data: Studies were required to report, or allow for the calculation of, diagnostic 2x2 contingency table data, including counts for true positives, false positives, false negatives, and true negatives.

Articles were excluded based on the following criteria: 1) Conference abstracts, case reports, reviews, commentaries, or editorials; 2) Unavailable full texts or incomplete data (after attempts to contact the corresponding authors were unsuccessful); 3) Studies with a sample size of fewer than 10 patients; 4) Studies that failed to distinguish between central compartment and lateral neck lymph node metastasis data; 5) Duplicate publications (only the version with the most comprehensive or most recent data was retained).

### 2.4 Literature screening and data extraction

Following the database search, all identified records were consolidated and imported into EndNote X9 to eliminate duplicates. The selection of eligible studies followed a two-stage, independent screening protocol. In the first stage, two researchers independently evaluated the titles and abstracts of all retrieved citations to exclude obviously non-relevant publications. In the second stage, the full texts of all remaining potentially relevant articles were procured. The same two reviewers then independently conducted a thorough evaluation of each full-text article, applying the pre-defined inclusion and exclusion criteria. Any disagreement regarding the eligibility of a study at either stage was addressed first through discussion between the two reviewers. If consensus could not be reached, the judgment of a third senior investigator was sought for final adjudication.

For each study that met the inclusion criteria, data were extracted independently by the same two researchers using a standardized, pre-piloted data collection form. After independent extraction, the two sets of extracted data underwent a cross-verification process to identify and rectify any inconsistencies, thereby ensuring accuracy. The information extracted included:

1. Study identification and design: First author, year of publication, country where the study was conducted, and the study design (e.g., retrospective cohort, prospective observational).
2. Participant Demographics and Methodology: Total sample size, demographic data (reported as mean age with standard deviation, or median with range), sex distribution, and specifics of the pre-operative calcitonin measurement assay employed.
3. Diagnostic accuracy metrics: The precise calcitonin concentration threshold(s) utilized in the study for predicting LLNM. For each reported threshold, the corresponding numbers of true positive (TP), false positive (FP), false negative (FN), and true negative (TN) cases were extracted, allowing for the construction of 2x2 contingency tables. If a single study presented results for multiple thresholds, the cut-off value identified as optimal by the study authors based on receiver operating characteristic (ROC) curve analysis was prioritized for extraction.

### 2.5 Assessment of methodological quality

The methodological quality and potential bias of each included study were assessed using the Quality Assessment of Diagnostic Accuracy Studies 2 (QUADAS-2) tool. This instrument offers a structured framework for evaluating bias across four key domains: (1) patient selection, (2) the index test (preoperative calcitonin measurement), (3) the reference standard (histopathological findings following neck dissection), and (4) flow and timing of study participants. Additionally, the tool assesses the applicability of the first three domains to the specific research question of this review. Two reviewers independently conducted the quality assessment for all included studies. Any disagreements regarding risk of bias or applicability concerns were resolved through discussion until a consensus was reached.

### 2.6 Statistical analysis

The statistical analysis was conducted in two parts. First, patients were stratified into two groups based on post-operative pathology: those with LLNM (LLNM-positive) and those without LLNM (LLNM-negative). To analyze the disparity in preoperative Ctn concentrations between the patient groups, a comparative statistical approach was adopted. For investigations that reported data using medians accompanied by interquartile ranges (IQR) or specific percentiles (such as the 25th and 75th percentiles), the validated methodologies outlined by Luo et al. (9) and Wan et al. (10) were implemented. These techniques allow for the estimation of the sample mean and standard deviation (SD) from reported non-parametric summary statistics, thereby enabling the inclusion of such studies in parametric meta-analytic models. Given the anticipated deviations from a normal distribution in biomarker levels and the inherent heterogeneity introduced by differing Ctn assay platforms and protocols across studies, a random-effects model utilizing the DerSimonian and Laird method was selected for synthesizing the effect estimates. The magnitude of the difference in preoperative Ctn levels between groups with and without LLNM was expressed using the standardized mean difference (SMD), accompanied by its corresponding 95% confidence interval (CI).

Second, using the extracted or derived 2x2 contingency table data, meta-analyses of diagnostic accuracy measures were performed. The meta-analysis of diagnostic performance was performed by calculating summary estimates, including pooled sensitivity, pooled specificity, and the diagnostic odds ratio (DOR). All estimates were derived with their associated 95% CIs through the application of a bivariate random-effects model. To synthesize the overall diagnostic accuracy across studies, a hierarchical summary receiver operating characteristic (HSROC) curve was generated, and the area under this curve (AUC) along with its 95% CI was determined. In accordance with the pre-defined analytical plan, subgroup analyses were executed to examine the influence of differing reported Ctn threshold values, such as comparisons between ≥300 pg/mL and 200 pg/mL, on the observed results and to investigate potential contributors to heterogeneity. The degree of statistical heterogeneity across the included studies was quantified using the I² statistic.

All statistical analyses and generation of forest plots, funnel plots, and the HSROC curve were performed using R software (version 4.3.0), primarily utilizing the ‘meta’ (version 6.5-0) and ‘mada’ (version 0.5.11) packages. A two-sided p-value of less than 0.05 was considered statistically significant.

## 3 Results

### 3.1 Literature screening process

**Figure 1:**
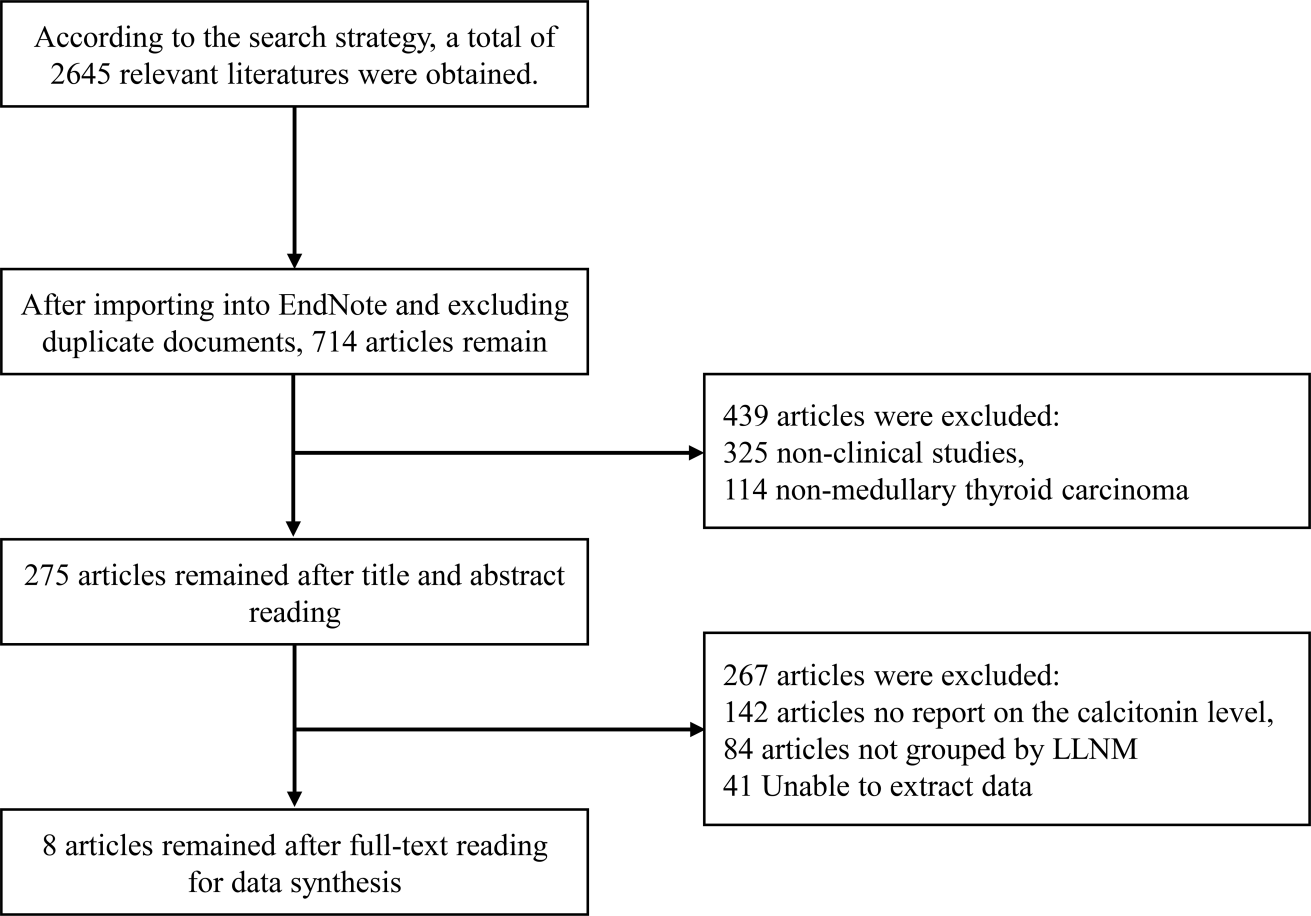
PRISMA flow diagram of literature selection.

The initial electronic database search yielded a total of 2,645 records. After removing duplicate entries using EndNote software, 714 unique citations were retained for further screening. This pool consisted of 562 English-language publications and 152 Chinese-language publications. During the initial screening phase based on titles and abstracts, 325 non-clinical records were excluded, along with an additional 114 publications not directly relevant to MTC. As a result, 275 articles were selected for full-text eligibility assessment. A thorough evaluation of these manuscripts against the predefined inclusion and exclusion criteria led to the exclusion of a further 267 studies. The final synthesis, therefore, incorporated a total of eight studies that satisfied all eligibility requirements for inclusion in the meta-analysis. A complete and sequential depiction of this study selection procedure is presented in the PRISMA flow diagram (**Figure 1**).

### 3.2 Basic characteristics of included studies

This meta-analysis ultimately incorporated 8 studies published between 2018 and 2025, encompassing a total of 951 patients. Seven of these studies were retrospective cohort designs (six from China and one from Korea), and one was an observational study from the United States. Regarding patient age, five studies reported data as mean ± standard deviation, with mean ages ranging from 48.16 to 51.38 years. The remaining three studies presented age data using stratification nodes of either 45 or 55 years. Gender distribution showed an imbalance in most studies, with only the study by Zhu J et al. (11) reporting an equal number of male and female patients (76/76). The Newcastle-Ottawa Scale (NOS) quality assessment scores for the included studies ranged from 6 to 8, indicating an overall quality level of moderate to high. (**Table 1**).

**Table 1:** Basic characteristics of included studies.

Author	Year	Country	Study design	Patients (n)	Age	Sex (M/F)	NOS
Zhao J (12)	2025	China	Retrospective	208	48.2 ± 12.8	99/109	7
Chen X (13)	2025	China	Retrospective	82	≤55 y: 57 >55 y: 25	38/44	8
Shi YF (14)	2020	China	Retrospective	39	<45 y: 19 ≥45 y: 20	13/26	6
Zhu J (11)	2024	China	Retrospective	152	48.16 ± 13.28	76/76	8
Ye L (15)	2020	China	Retrospective	74	51.38 ± 12.89	39/35	7
Park H (16)	2021	Korea	Retrospective	246	49.4 ± 14.5	60/112	7
Lu QC (17)	2019	China	Retrospective	84	<45 y: 23 ≥45 y: 61	30/54	6
Pena I (18)	2018	American	Observational	66	55(25-85)	25/41	7

### 3.3 Difference in pre-operative calcitonin levels between LLNM and non-LLNM groups

**Figure 2:**
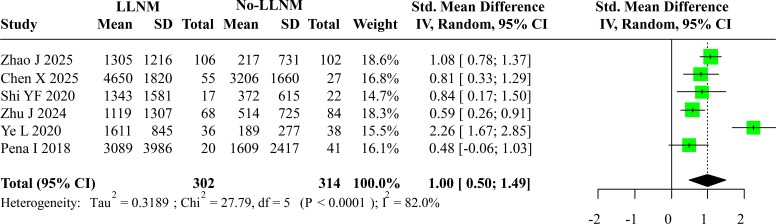
Forest plot of preoperative calcitonin levels in LLNM vs. non-LLNM groups.

**Figure 3:**
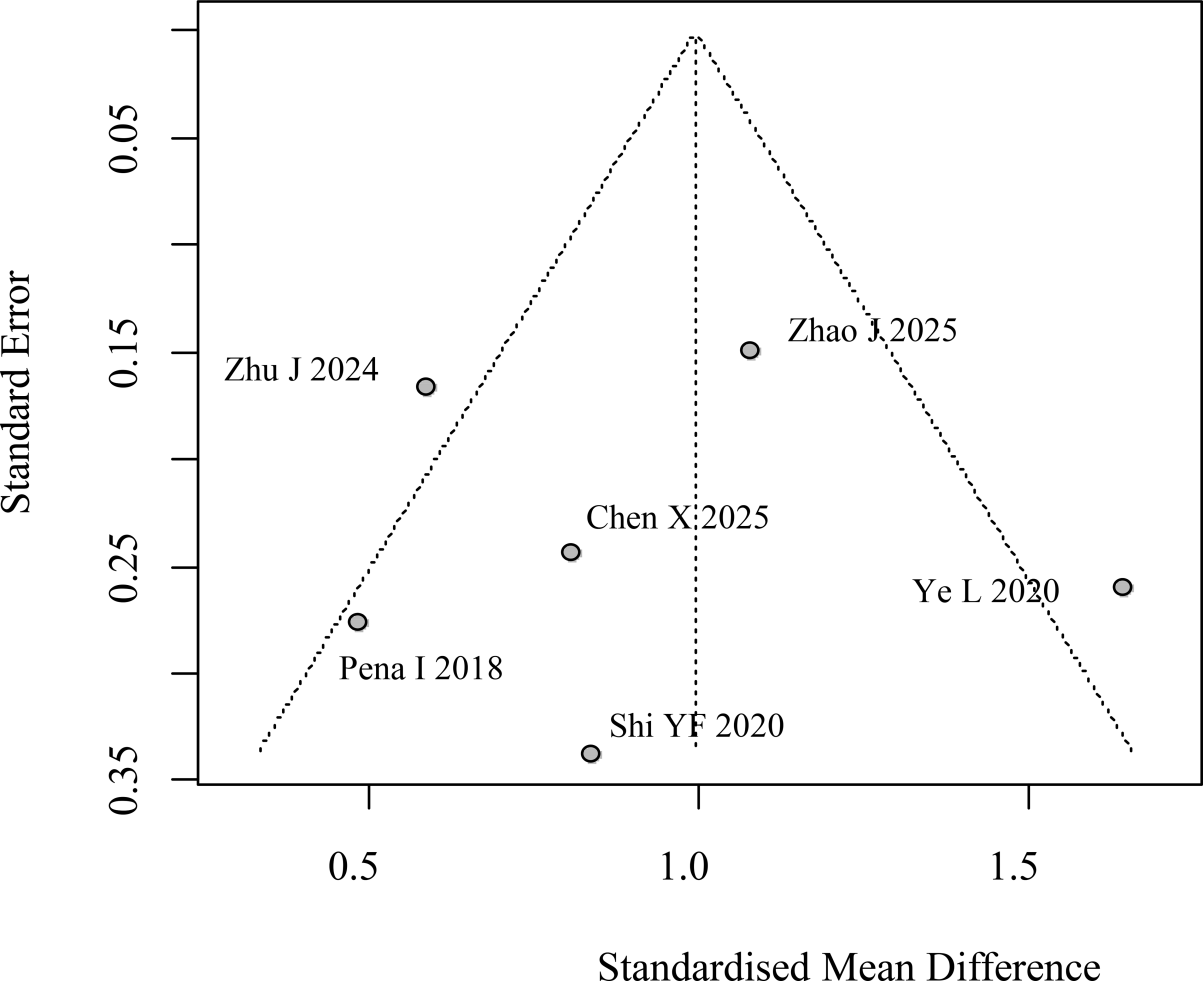
Funnel plot for assessment of publication bias in preoperative calcitonin levels between LLNM and non-LLNM groups.

Six studies reported pre-operative calcitonin levels for both LLNM and non-LLNM patient groups. Due to considerable heterogeneity detected across the included studies, a random-effects model was selected to synthesize the effect estimates. This approach accounts for variability beyond chance alone. To mitigate the confounding influence of disparate measurement protocols and assay platforms, the SMD was chosen as the primary effect measure for the comparative analysis. The pooled results revealed a statistically significant elevation in preoperative Ctn levels among patients with confirmed LLNM relative to those without metastasis. The summary effect size was an SMD of 1.00 (95% Confidence Interval: 0.50 to 1.49), indicating a substantial difference. A forest plot detailing the individual and pooled estimates from each contributing study is provided in **Figure 2**. Given the modest number of studies (n=6) available for this analysis, a formal statistical test for publication bias was not performed. Instead, potential bias was evaluated through visual inspection of a funnel plot (**Figure 3**). The symmetrical distribution of study points observed in the funnel plot suggests a low likelihood of significant publication bias affecting these findings.

### 3.4 Diagnostic performance of pre-operative calcitonin level for LLNM

Six studies provided data on the diagnostic performance of pre-operative calcitonin for LLNM. Based on the reported cut-off values, these studies were categorized into two subgroups for analysis: a 200 pg/mL subgroup and a high-threshold subgroup (designated as ≥300 pg/mL, with reported cut-offs ranging from 300 to 317 pg/mL), each containing three studies.

**Figure 4:**
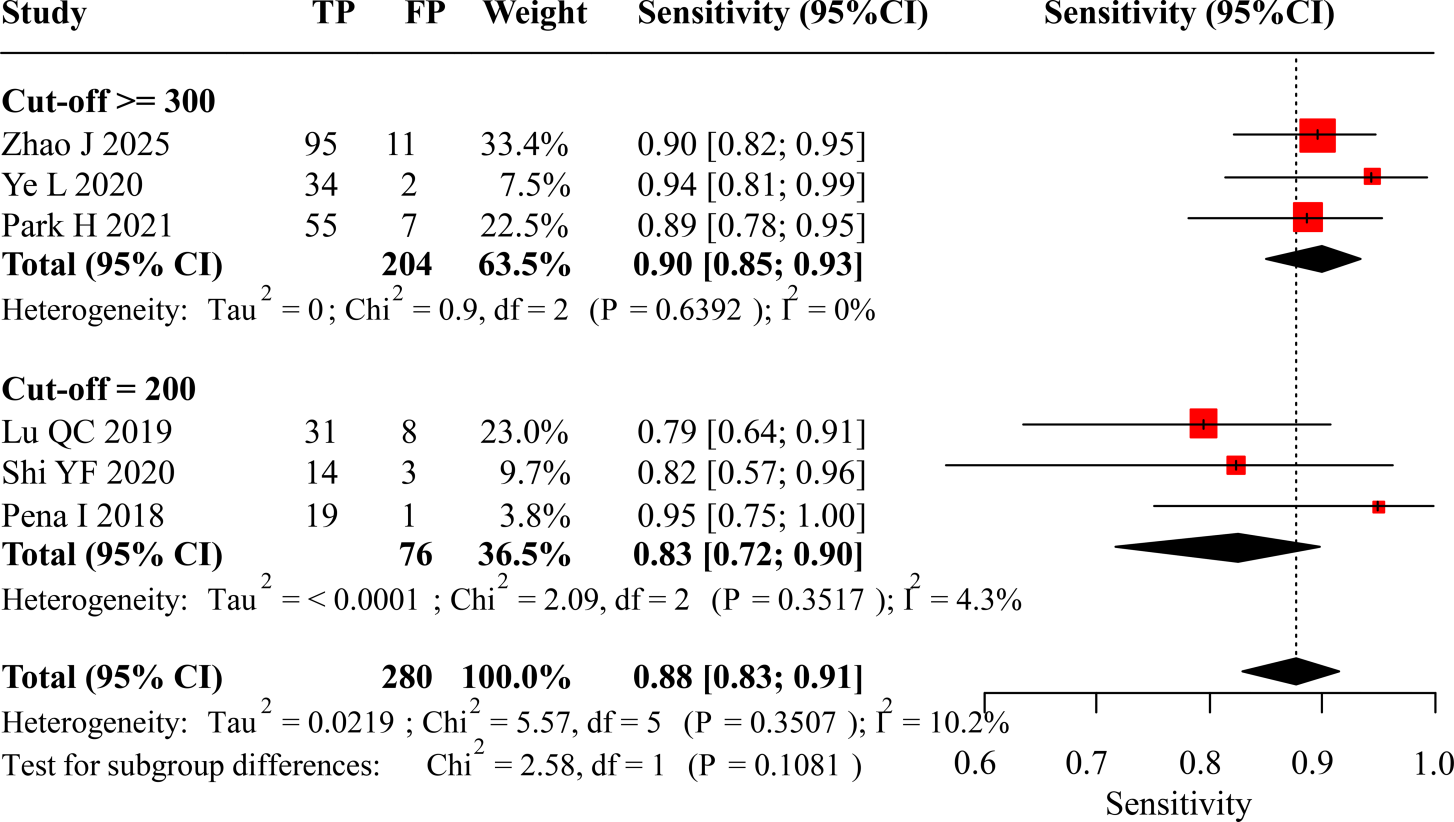
Forest plot of sensitivity for preoperative calcitonin in diagnosing LLNM.

Regarding diagnostic sensitivity, the meta-analytic synthesis for studies employing a calcitonin threshold of ≥300 pg/mL yielded a pooled estimate of 0.90 (95% CI: 0.85 to 0.93). Conversely, analyses restricted to studies using the lower 200 pg/mL cut-off produced a pooled sensitivity of 0.83 (95% CI: 0.72 to 0.90). When data from all studies were combined, irrespective of the specific threshold, the overall pooled sensitivity was 0.88 (95% CI: 0.83 to 0.91). These results show a nominally higher pooled sensitivity in the ≥300 pg/mL subgroup (0.90) compared to the 200 pg/mL subgroup (0.83). While a lower threshold typically improves sensitivity, this counterintuitive trend was not statistically significant (P = 0.1081) and likely reflects the inherent clinical and methodological heterogeneity among the limited number of studies in each subgroup. The corresponding forest plot illustrating these sensitivity estimates is displayed in **Figure 4**.

**Figure 5:**
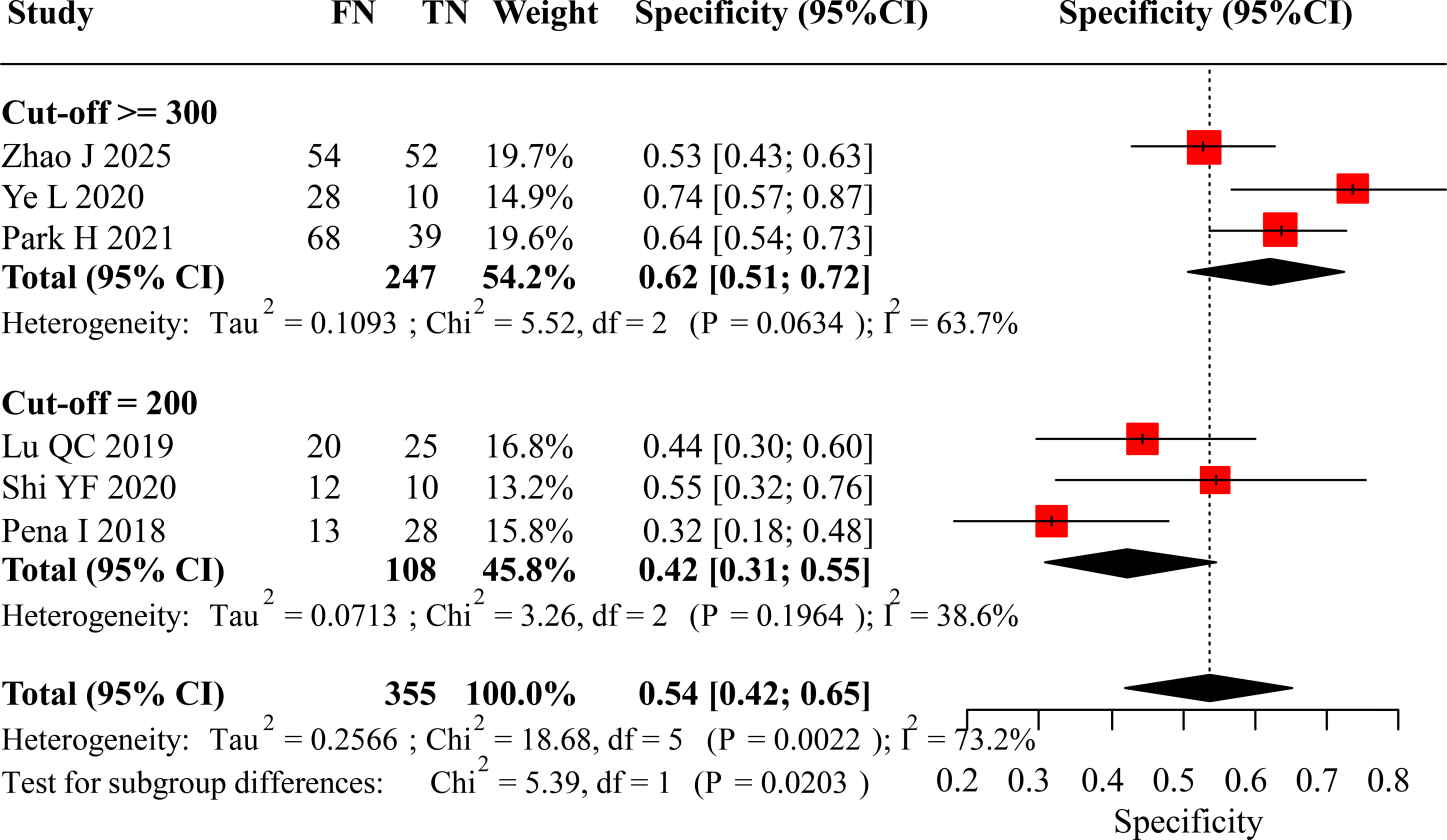
Forest plot of specificity for preoperative calcitonin in diagnosing LLNM.

Analysis of diagnostic specificity revealed a distinct pattern. The pooled specificity for the ≥300 pg/mL subgroup was 0.62 (95% CI: 0.51 to 0.72). In contrast, the pooled specificity for the 200 pg/mL subgroup was markedly lower at 0.42 (95% CI: 0.31 to 0.55). The overall pooled specificity across all studies was 0.54 (95% CI: 0.42 to 0.65). A statistical comparison confirmed that the specificity associated with the ≥300 pg/mL threshold was significantly superior to that of the 200 pg/mL threshold (p = 0.0203). The forest plot detailing these specificity calculations is provided in **Figure 5**.

**Figure 6:**
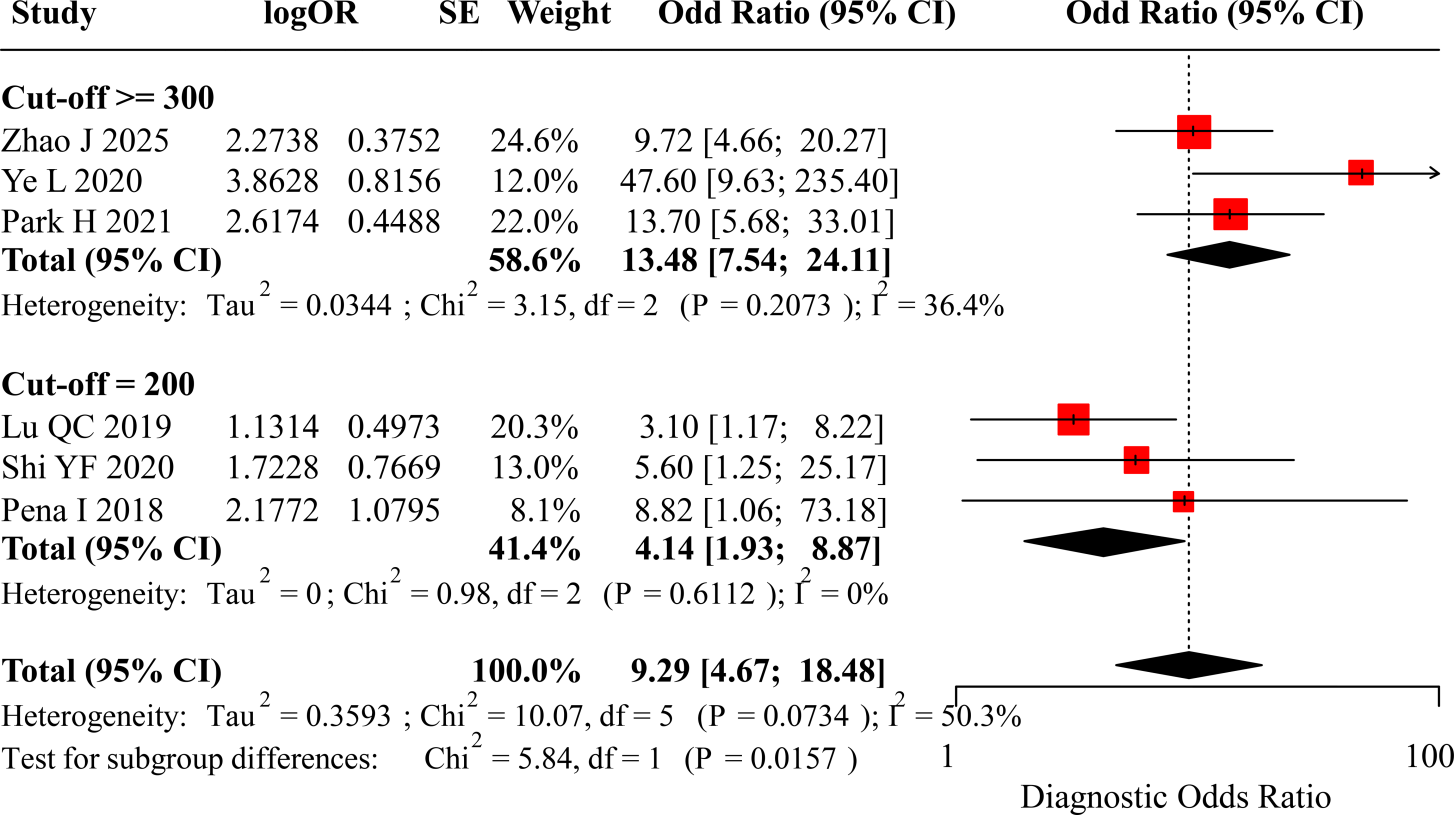
Forest plot of diagnostic odds ratio (DOR) for preoperative calcitonin in diagnosing LLNM.

**Figure 7:**
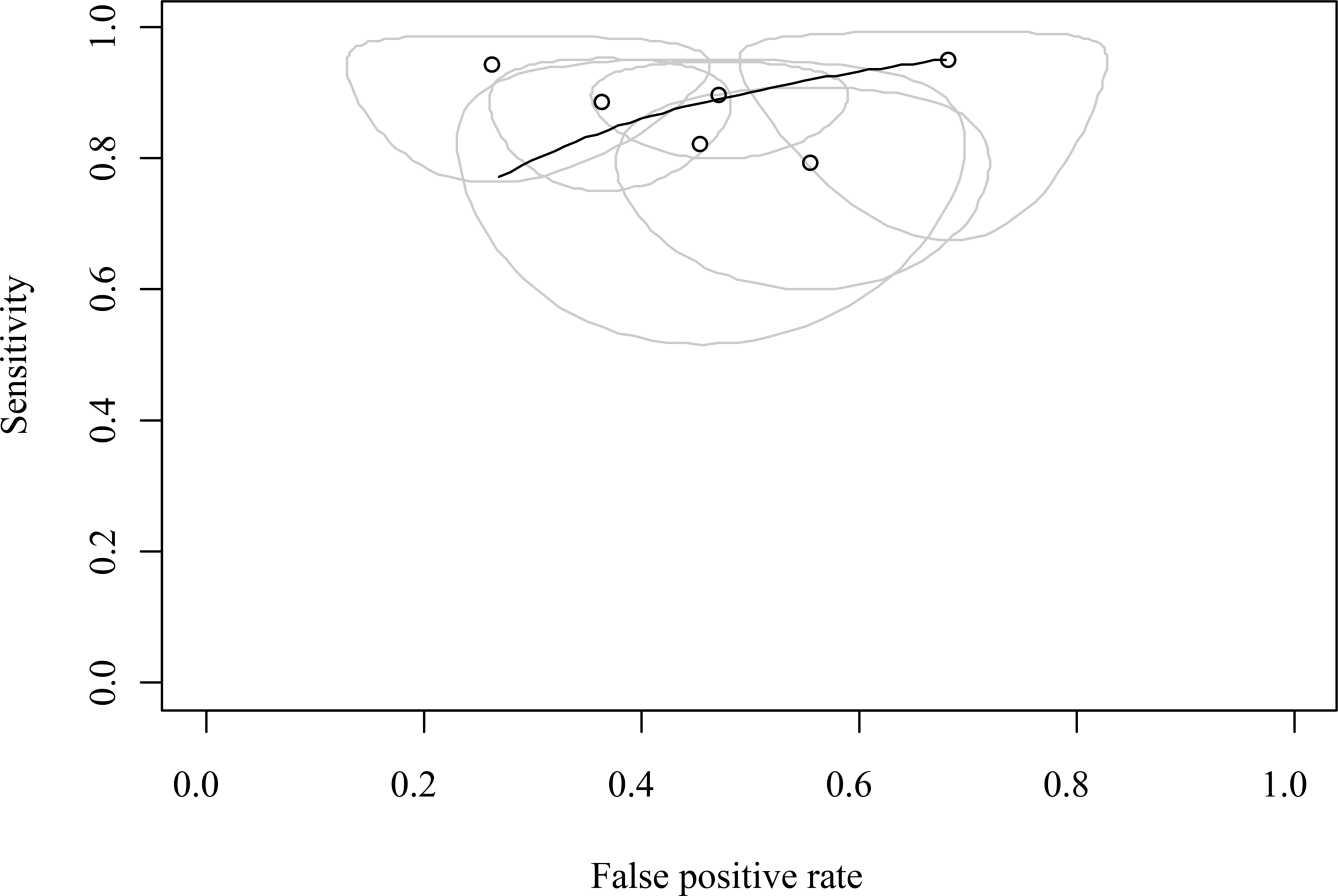
Summary receiver operating characteristic (SROC) curve for preoperative calcitonin in diagnosing LLNM.

For the diagnostic odds ratio (DOR), the pooled DOR for the Cut-off ≥300 subgroup was 13.48 (95% CI: 7.54, 24.11). The pooled DOR for the Cut-off 200 subgroup was 4.14 (95% CI: 1.93, 8.87). The overall pooled DOR was 9.29 (95% CI: 4.67, 18.48). The DOR was significantly higher in the Cut-off ≥300 subgroup compared to the Cut-off 200 subgroup (p = 0.0157). The forest plot for DOR is displayed in **Figure 6**. The area under the summary receiver operating characteristic (SROC) curve was 0.894 (95% CI: 0.834, 0.965), as illustrated in **Figure 7**.

## 4 Discussion

This systematic review and meta-analysis provides the first quantitative synthesis of evidence demonstrating the substantial predictive value of preoperative serum Ctn for detecting LLNM in MTC. At a threshold of ≥300 pg/mL, the pooled estimates yielded a diagnostic sensitivity of 0.90, specificity of 0.62, DOR of 13.48, and an SROC-AUC of 0.894. Collectively, these metrics constitute high-level evidence to inform individualized surgical planning for cervical lymphadenectomy. Notably, although MTC accounts for only 1–2% of all thyroid malignancies, it contributes disproportionately to disease-specific mortality, representing 8–13% of thyroid cancer-related deaths (19). LLNM is the primary determinant of postoperative persistence or recurrence and critically influences both disease-free survival and the likelihood of reoperation (20). The clinical utility of calcitonin in guiding surgical decision-making is increasingly recognized. An individualized approach to lymphadenectomy based on calcitonin levels may preserve oncologic efficacy while reducing the risk of surgical complications (3).

Conventional imaging modalities exhibit limited sensitivity for detecting micrometastases. This study demonstrates that a preoperative Ctn threshold of ≥300 pg/mL captures patients at high risk for LLNM with 90% sensitivity, thereby establishing a quantitative benchmark for “serum-driven” selective neck dissection. The designation of preoperative calcitonin as a core risk factor for MTC-LLNM (SMD = 1.39, P<0.00001) in a recent meta-analysis by Lin et al. (21) corroborates its pivotal role in metastasis prediction (21). Du et al. (22), based on a multicenter cohort, proposed a refined cut-off of 693.9 pg/mL for ipsilateral LLNM, identifying calcitonin as the most sensitive and specific serum marker for MTC. Their proposed threshold was associated with significantly improved structural recurrence-free survival compared to older ATA benchmarks. Synthesizing data from multiple sources, the present analysis suggests that a threshold of 300 pg/mL is pragmatically sufficient for clinical decision-making in real-world settings with variable resources, while centers with advanced assay capabilities may benefit from adopting a more granular threshold like 693.9 pg/mL to achieve “stratified precision.” This highlights that optimal Ctn thresholds may require dynamic adjustment based on the specific metastatic pattern and clinical context. Furthermore, the SROC-AUC of 0.894 observed in this study is comparable to the AUC of 0.890 reported by Ye et al. (15) using a panel combining Ctn, CEA, and NSE, indicating that single-marker Ctn testing already possesses substantial diagnostic accuracy, offering a practical alternative in resource-constrained environments.

The analysis revealed moderate to substantial heterogeneity, particularly for specificity (I² = 63.7%), while heterogeneity for sensitivity was low (I² = 10.2%). This heterogeneity likely stems from several sources. First, variability in detection methodologies is a key contributor. As noted by Du et al. (23), differences in assay precision between techniques such as electrochemiluminescence and chemiluminescence can lead to fluctuations in established cut-off values. The included studies did not employ a uniform assay method or standardized cut-off values, representing a significant source of heterogeneity. Notably, the internal variation within the high-threshold subgroup (ranging from 300 to 317 pg/mL) is not negligible and may further contribute to the observed statistical variance within this category. Second, differences in patient population characteristics may have influenced the results. Seven of the eight included studies were conducted in Chinese populations, with one Korean cohort by Park et al. (24). Since clinical features such as gender and tumor diameter have been correlated with LLNM risk (25), variations in gender ratios, age distributions, and tumor stages across studies could affect result consistency. Third, inconsistencies in lymph node assessment standards may introduce bias. While studies failing to distinguish between central and lateral compartment metastasis data were excluded, differences in the extent of lymph node dissection and the granularity of pathological evaluation among the included studies, as emphasized by Lin et al. (21), could further contribute to heterogeneity. A noteworthy finding in our subgroup analysis was the paradoxically lower sensitivity observed at the 200 pg/mL threshold compared to the ≥300 pg/mL threshold (0.83 vs 0.90). Theoretically, lowering the diagnostic cutoff should increase the true positive rate; however, our results may be influenced by differences in study-specific populations or assay platforms. For instance, studies in the 200 pg/mL subgroup might have included a higher proportion of patients with micro-metastases or lower tumor volumes, which are inherently more difficult to detect regardless of the cutoff utilized. Additionally, the small number of studies (n=3 per subgroup) increases the impact of individual study outliers on the pooled estimates. This suggests that while a threshold of 300 pg/mL appears robust, the choice of an ‘optimal’ cutoff must consider the specific assay performance and patient risk profile of the clinical setting.

The findings possess clear translational clinical value. LLNM status in MTC directly dictates surgical extent and prognosis (4), yet traditional imaging has limited diagnostic performance for micrometastases. Serum calcitonin serves as a non-invasive biomarker capable of compensating for this imaging shortfall. When preoperative Ctn is ≥300 pg/mL, the high sensitivity (0.90) effectively identifies patients at elevated risk for metastasis, warranting consideration of lateral neck dissection. For patients with Ctn <200 pg/mL, although sensitivity is somewhat lower (0.83), this level, in conjunction with ultrasonography (26), may help avoid unnecessary surgical morbidity. Furthermore, aligning with the paradigm of “risk-oriented” surgery for MTC advocated by Machens et al. (27), the calcitonin thresholds defined herein provide a quantitative foundation for tailoring surgical plans. This is particularly relevant for patients who are clinically node-negative (cN0) but exhibit elevated calcitonin, aiding in the prevention of both overtreatment and undertreatment.

The interpretation of our findings should be considered in light of several methodological constraints. A primary limitation is the modest total of studies (n=8) meeting the eligibility criteria for inclusion in this meta-analysis, and the preponderance of retrospective designs (7 studies) introduces the potential for selection and reporting biases. Second, the analysis was restricted to basal calcitonin levels; stimulated calcitonin values, which may enhance diagnostic accuracy as suggested by Niederle et al. (28), were not incorporated, potentially underestimating the full diagnostic potential of the marker. Third, molecular characteristics such as RET mutation status were not considered. Given that RET mutations correlate with both calcitonin levels and metastatic risk (29), future analyses would benefit from stratifying patients based on such genetic variables. Fourth, the lack of a standardized assay protocol across studies means that differences in laboratory-specific reference ranges may affect the generalizability of the proposed cut-off values.

## 5 Conclusion

Preoperative serum calcitonin at a threshold of ≥300 pg/mL serves as a reliable, non-invasive indicator for predicting lateral cervical lymph node metastasis in patients with medullary thyroid carcinoma. It provides high-level evidence to support “risk-oriented,” individualized neck dissection strategies. For optimal clinical translation, it is recommended that this biomarker be integrated with ultrasonographic features and mutational profiling to facilitate composite decision-making, thereby refining surgical extent and minimizing the risks associated with overtreatment.
